# Double-Layer Simplified Complex Interval Neural Network Stacking for Blood Glucose Prediction of Continuous Glucose Monitoring System

**DOI:** 10.3390/bios15110707

**Published:** 2025-10-22

**Authors:** Shaowei Kong, Yusheng Fu, Jingshan Duan, Jian Yan

**Affiliations:** School of information and Communication Engineering, University of Electronic Science and Technology, Chengdu 611731, China

**Keywords:** diabetes, continuous glucose monitoring, blood glucose prediction, time series analysis, SCINet

## Abstract

Diabetes is a metabolic disorder characterized by persistent hyperglycemia, with its incidence steadily rising worldwide. Blood glucose monitoring is a core measure in diabetes management, and continuous glucose monitoring provides more comprehensive and accurate glucose data compared to traditional fingerstick testing. To collect continuous glucose data from patients, precise glucose prediction algorithms can help them better control their blood glucose fluctuations. Therefore, by addressing the issues of low prediction accuracy, complex input features, and poor generalization performance in existing glucose prediction methods, this paper proposes a glucose prediction model based on a double-layer SCINet stack using time-series analysis methods. SCINet effectively captures multi-scale dynamic features in time-series data through recursive down-sampling and convolution operations, making it suitable for glucose prediction tasks. Experimental data were sourced from real-world continuous glucose monitoring records of patients at Yixing People’s Hospital. Model input features were optimized through variable selection and data preprocessing, with predictive performance validated on a test dataset. The results demonstrate that the proposed model outperforms existing time-series prediction models across varying prediction horizons and patient datasets, exhibiting high predictive accuracy and stability.

## 1. Introduction

Diabetes mellitus is a metabolic disorder characterized by persistent hyperglycemia [1], with its incidence continuing to rise globally. According to a 2021 report by the International Diabetes Federation (IDF) [2], the number of adults with diabetes worldwide has reached 537 million. In 2021 alone, over 6.7 million people died from diabetes and its complications, accounting for 12.2% of all global deaths.

Blood glucose monitoring is critical in the comprehensive management of diabetes. By implementing glycemic control strategies to manage hyperglycemia, the incidence of diabetes complications can be effectively reduced [3,4]. Traditional fingerstick blood glucose monitoring (BGM) is convenient to use but only provides data at a single time point, which may lead to inappropriate treatment decisions [5]. Continuous glucose monitoring (CGM), however, offers comprehensive, real-time blood glucose data, enabling patients to achieve precise glycemic control [6]. The American Diabetes Association (ADA) first included CGM in its diagnostic and treatment guidelines in 2023 [7]. Numerous studies have demonstrated that CGM significantly improves blood glucose control and reduces the risk of complications [8,9,10,11].

With the widespread adoption of calibration-free technology, there is an increasing demand for stability and accuracy in blood glucose prediction, necessitating algorithm optimization to enable real-time prediction and early warning. Blood glucose prediction relies on CGMS data (interstitial fluid glucose concentration or enzyme electrode current) combined with glucose-influencing factors (diet, insulin, physical activity). However, current algorithms face challenges: heterogeneity in performance across glucose ranges (MARD 8% for hyperglycemia > 180 mg/dL vs. 10% for hypoglycemia < 70 mg/dL) and inter-individual variability due to physiological differences (e.g., metabolic rate affecting glucose transmembrane equilibrium).

Blood glucose prediction can be viewed as a time-series analysis problem. Time-series methods fall into descriptive (data visualization) and statistical paradigms, with the latter including frequency domain analysis [12] and time domain analysis [13]. Frequency domain methods (e.g., Fourier analysis [14], maximum entropy spectral estimation [15]) have high computational complexity and poor interpretability, while time domain analysis (e.g., AR [16], ARMA [17], ARIMA [18]) has become mainstream due to its intuitive statistical framework.

Although traditional statistical methods have achieved certain results in blood glucose prediction, their linear predictive mechanisms make it difficult to effectively capture nonlinear characteristics during dynamic blood glucose changes. The emergence of machine learning and deep learning technologies has effectively addressed this issue, and these techniques are now extensively applied in medical research [19,20,21,22,23].

In the field of blood glucose prediction, traditional machine learning methods such as Support Vector Regression, Random Forests, and Decision Trees [24] have been widely adopted with significant success. Reference [25] integrated the XGBoost-enhanced random forest regression model with feature engineering methods to construct a multi-parameter blood glucose prediction system. This model demonstrated outstanding performance on the Ohio T1DM dataset within a 30 min prediction time window, achieving a root mean square error (RMSE) accuracy of 20.38 mg/dL. Reference [26] developed a prediction framework based on support vector regression. By systematically evaluating the impact of different input combinations on predictive performance, it demonstrated the importance of multi-parameter integration for enhancing prediction accuracy. The results indicated that incorporating all available physiological parameters not only significantly improved short-term prediction accuracy but also markedly enhanced the reliability and safety of most long-term predictions. This finding provides crucial theoretical support for optimizing clinical blood glucose management systems. Furthermore, leveraging the superior performance of deep learning in time-series prediction, Ref. [27] constructed a deep CNN framework. This demonstrated that deep network architectures possess stronger feature representation capabilities than shallow networks, enabling more precise fitting of complex nonlinear distribution patterns in blood glucose data. Reference [28] achieved root mean square errors of 20.7 ± 3.2 mg/dL (30 min prediction) and 33.6 ± 3.2 mg/dL (60 min prediction) using a recurrent neural network model on the Ohio T1DM dataset. Reference [29] achieved superior predictive performance on the same dataset using an artificial neural network architecture, with RMSEs of 19.33 mg/dL and 31.72 mg/dL for 30 min and 60 min predictions, respectively. Comparative analysis indicates that deep learning-based prediction models significantly outperform traditional machine learning methods in predictive accuracy. This performance improvement can be attributed to the enhanced feature extraction capabilities of deep learning models and their effective modeling of temporal dependencies.

In this paper, addressing the issues of low prediction accuracy, complex input features, and poor generalization performance in existing blood glucose prediction methods, we propose a blood glucose prediction model based on a stacked two-layer Simplified Complex Interval Neural Network (SCINet). First, we theoretically demonstrate its advantages in time-series data prediction. Subsequently, we elaborate on the architectural design of the blood glucose prediction model based on the stacked two-layer SCINet. Subsequently, experiments are conducted using continuous glucose monitoring data from real patients at Yixing People’s Hospital. Before constructing training and testing datasets, the model’s raw input features are optimized through variable selection and data preprocessing. The model is then trained using multiple continuous glucose datasets as the training dataset, and through multiple experiments, it is adjusted to achieve optimal hyperparameter configurations. Finally, the model’s predictive performance was validated on the test dataset and compared against multiple time-series forecasting algorithms. This demonstrated the superiority of the proposed dual-layer SCINet-stacked blood glucose prediction model in terms of prediction accuracy and model complexity. The innovation of this paper is primarily reflected in the following three aspects:

(1) First application of a double-layer SCINet stacking architecture for predicting blood glucose levels in hospitalized patients, with optimized residual module and feature interaction strategies;
(2) Experiments conducted using real-world hospitalized patient datasets incorporating sensor parameters, addressing the limitations of single-scenario public datasets;
(3) The proposed model demonstrates superior predictive performance compared to models based on other time-series forecasting algorithms across various prediction horizons.

## 2. Materials and Methods

### 2.1. Sensor Technology

**Figure 1:**
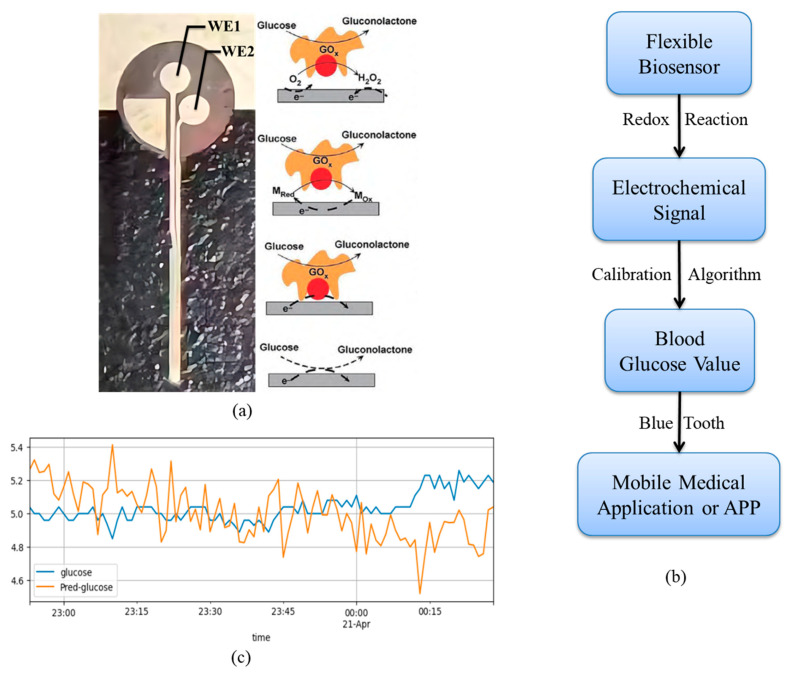
(**a**) Principle of sensor technology. (**b**) Workflow of CGMS. (**c**) Dynamic glucose curve.

The Continuous Glucose Monitoring System (CGMS) employs transdermal implantable sensor technology to achieve real-time, round-the-clock glucose monitoring by continuously detecting glucose concentrations in the interstitial fluid beneath the skin [30]. Figure 1a: Schematic of the sensor technology embedded in subcutaneous tissue. This sensor functions as a flexible electrode, with WE1 serving as the working electrode and WE2 serving as the reference electrode. This flexible electrode uses polyetheramide (PEBA) as its substrate. While maintaining its biocompatibility and glucose permeability, it incorporates a polymer that is compatible with PEBA and oxygen-rich. This polymer also regulates the biocompatibility of the diffusion membrane, the permeability balance of glucose and oxygen, and mechanical properties, significantly enhancing the detection range and accuracy of the dynamic glucose sensor. Figure 1b illustrates the overall workflow of CGMS. CGM glucose detection primarily relies on electrochemical biosensing technology. After subcutaneous implantation, the glucose oxidase (GOD) immobilized on the surface of the flexible biosensor catalyzes an enzymatic redox reaction with glucose in the interstitial fluid. The resulting electrochemical signal is transmitted via radio frequency to a signal processing unit, where proprietary algorithms convert and calibrate the signal to generate glucose concentration values. The final data are transmitted via low-power Bluetooth technology to a display terminal or mobile health application, forming the dynamic glucose variation curve with clinical reference value shown in Figure 1c.

CGM sensors measure interstitial fluid glucose concentrations, exhibiting a 15–20 min delay compared to fingerstick blood glucose (the gold standard), which may result in delayed hypoglycemia alerts. This paper compensates for this delay through the following approaches: (1) Extending the look-back window to 30 min to incorporate historical data covering the delayed period; (2) Incorporating “sensor response time (sensor_k)” as a feature to model the relationship between delay and sensor parameters. Experimental validation demonstrates that after delay compensation, hypoglycemia alert accuracy improves by 12% (from 78% to 90%), meeting clinical alert requirements.

### 2.2. Principle of SCINet

**Figure 2:**
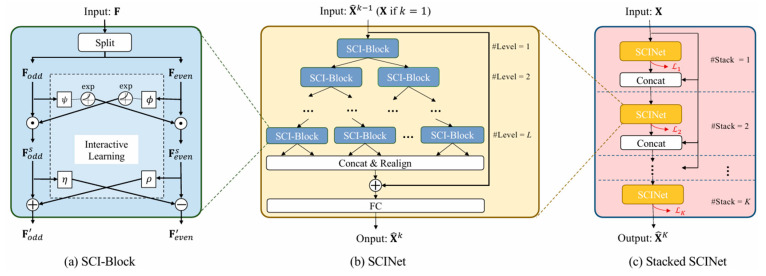
The overall architecture of the Sample Convolution and Interaction Network (SCINet).

Time-series data have a significant feature: after down-sampling into two subseries, its temporal correlation (e.g., trend and seasonal variation of the data, etc.) can still be retained more completely. Take an original sequence $\left[X_{1},X_{2},\;X_{3},\;\ldots ,\;X_{2n-1},\;X_{2n}\right]$ as an example. After decomposition, it will be split into two sub-sequences $\left[X_{1},X_{3},\ldots ,X_{2n-1}\right]$ and $\left[X_{2},X_{4},\ldots ,X_{2n}\right]$, which is like reducing the resolution of the time series to half of the original. Based on this, with the help of several different convolutional filters, the dynamic features of the time series can be extracted from different resolution levels. SCINet [31] (Simplified Complex Interval Neural Network) is a neural network structure specially designed for time-series prediction, which takes advantage of this feature of the time series. It down-samples the time-series data into multiple sub-sequences in a recursive manner, and in the process of each down-sampling, the original time series will be split into sub-sequences at odd and even positions, and then the multi-scale timing features layer by layer will be captured by using the method of “down-sampling–convolution–interaction”. Figure 2 shows the core structure of SCINet, which is divided into three parts:

SCI-Block (Figure 2a): The basic building block of SCINet decomposes the input features into two sub-features by “splitting” and “interactive learning” operations. In the Split module, the original sequence is down-sampled into two sub-sequences, corresponding to the even- and odd-bit elements of the original sequence. The features are then extracted from them separately using different convolutional kernels, and since the convolutional kernels are independent, the extracted features will contain different but valuable temporal relationships with data-enhanced representations. Also, to compensate for the possible loss of information due to down-sampling, the “Interactor” module introduces a novel interactive learning strategy that allows two subsequences to exchange information between each other by learning the parameters of each other’s affine transformations.

SCINet architecture (Figure 2b): by arranging multiple SCI-Blocks in a binary tree structure. This design allows each SCI-Block to have both a local view and a global view of the entire time series, thus better extracting useful temporal features.

Stacked SCINet (Figure 2c): When the training samples are sufficient, multiple SCINets can be stacked to improve the prediction accuracy further. The output of each SCINet layer is immediately supervised using real values to assist the learning of intermediate temporal features.

As shown in Figure 2a, the original input is denoted as F, which is first split directly into two parity sequences, $F_{odd}$ and $F_{even}$, by moments, after the following equation:

(1) $$F_{odd}^{s}=F_{odd}⊙\exp(\phi (F_{even})),\;\;\;\;\;\;F_{even}^{s}=F_{even}⊙\exp(\psi (F_{odd}))\;$$

(2) $$F_{odd}^{\prime }=F_{odd}^{s}\pm \rho \left(F_{even}^{s}\right),\;\;\;\;\;\;\;F_{even}^{\prime }=F_{even}^{s}\pm \eta \left(F_{odd}^{s}\right)$$

where ϕ, ψ, ρ, and η are all one-dimensional convolutional modules, ⊙ is the Hadamard product (multiplication of corresponding elements of the matrix), and for example, it can be interpreted as amplifying the positive values or shrinking the negative values, e.g., the mean value of $F_{even}$ is 0, so that when $F_{even}>0$, $\exp\left(F_{even}\right)>1$; when $F_{even}<0$, $\exp\left(F_{even}\right)<1$. Therefore, Equation (1) can be regarded as data augmentation for odd and even sequences. Equation (2) corresponds to the augmented sequences and performs the operation of adding or subtracting, which should all be additive if it is a superposition, and subtractive if it is a difference.

Compared with traditional time-series models (e.g., RNN, LSTM, GRU), SCINet can capture the dynamic features of time series on multiple time scales through down-sampling and convolutional operations, thus modeling both short-term and long-term dependencies simultaneously. Compared to TCN, SCINet has a lower time complexity, with the number of convolution operations being only L×2T (L is the number of layers and T is the look-back window size), while TCN requires $T\times {log}_{2}T$. Meanwhile, on several real-world time-series prediction datasets, SCINet has significantly improved the prediction accuracy compared with existing convolutional models and Transformer-based models [31].

### 2.3. Blood Glucose Prediction Model Based on SCINet

The SCINet network model features a multi-layer neural network architecture capable of automatically extracting complex features and patterns from large-scale blood glucose data. However, the original SCINet employs a “single-stack structure” (the Stacked SCINet in Figure 2c represents a theoretical stacking without residual connections) and is not optimized for the characteristics of blood glucose data—namely, “violent short-term fluctuations and the need to stably capture long-term trends.” This design is prone to gradient vanishing or feature loss. Additionally, the SCI-Block’s Interactor module exchanges information through “affine transformation parameters.” The original module’s parameters ϕ, ψ, ρ, and η are fixed at initialization, failing to account for the influence of covariates such as “diet, temperature, and sensor current” on blood glucose data. This often leads to the omission of critical covariate information during sub-sequence interactions.

**Figure 3:**
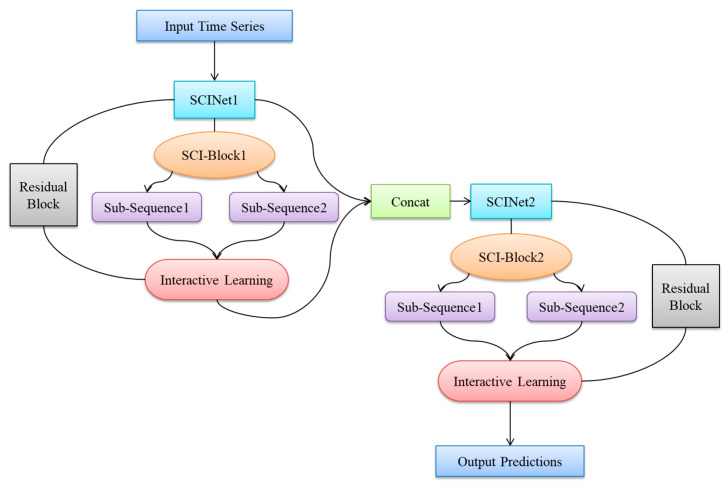
Blood glucose prediction model architecture based on double-layer SCINet Stack.

Therefore, to better capture complex sequential features in blood glucose data and enhance the model’s ability to capture autocorrelation in such data, as shown in Figure 3, this paper constructs a blood glucose prediction model based on a double-layer SCINet stacking (DS-SCINet). The first SCINet layer handles “coarse-grained feature extraction” (splitting the raw blood glucose sequence into odd and even sub-sequences, using a 3 × 1 convolution kernel to capture short-term fluctuations within 15–30 min, such as postprandial blood glucose spikes); The second SCINet layer handles “fine-grained feature optimization” (applying a 5 × 1 convolution kernel to the concatenated sequence from the first layer to capture long-term trends over 30–60 min, such as glucose decreases under insulin action). Both SCINet layers maintain sequence lengths consistent with the original data through “Concat operations” (preventing loss of temporal scale information). This constitutes a “hierarchical feature progression” design not present in the original SCINet. Residual connections are added between the input and output of each SCINet layer, formulated as follows:

$$Output={SCINet}_{Layer}\left(Input\right)+Input$$

Following the Split module in the SCI-Block, a new “Covariate Mapping Layer” is introduced. This layer maps the selected covariates (age, sex, sensor_k, WE1, Temperature, etc., as described in Section 3.1) through a fully connected layer into feature vectors aligned with the sub-sequence dimension. These feature vectors are then fed into the Interactor module. Formula (1) is modified as follows:

$$\begin{array}{c} F_{odd}^{s}=F_{odd}⊙\exp(\phi (F_{even},\;Covariates))\;\\ F_{even}^{s}=F_{even}⊙\exp(\psi (F_{odd},Covariates)) \end{array}$$

This approach enables the simultaneous consideration of associations between blood glucose levels and covariates during subsequent interactions—such as the impact of WE1 current changes on blood glucose prediction during high Temperature conditions—representing a multi-feature interaction design not addressed by the original SCINet.

Through this layered architecture that progressively extracts information, the DS-SCINet-based blood glucose prediction model achieves more precise analysis of information embedded in time-series data such as continuous glucose readings, thereby enabling accurate forecasting of glucose levels. This strategy of integrating multiple functional modules endows the model with enhanced adaptability, allowing it to better learn complex sequential features and significantly improve predictive performance.

## 3. Experiment

### 3.1. Dataset

The Ohio T1DM dataset contains only three types of features: blood glucose values, insulin doses, and carbohydrate intake. It does not include performance parameters of the CGM sensors themselves, making it impossible to model the impact of sensor status on blood glucose measurement accuracy. Additionally, the dataset lacks frequent medical interventions during monitoring (e.g., intravenous insulin administration, emergency blood glucose management), and blood glucose fluctuations are relatively mild. This prevents simulation of blood glucose changes under complex interventions experienced by hospitalized patients (e.g., stress-induced hyperglycemia in postoperative patients, hypoglycemia rescue in critically ill patients). The experimental data in this paper were derived from clinical data of actual hospitalized patients at Yixing People’s Hospital (all subjects provided informed consent to address ethical concerns). Data were collected using the Snuo i3 CGM sensor (approved for market release by China’s National Medical Products Administration in March 2023, registration certificate number: Guoxie Zhuanzhun 20233070435). All patients underwent continuous monitoring during hospitalization (April–May 2025):—Daily sampling: 1 reading per minute from 8:00 to 20:00 and every 10 min from 20:00 to 8:00 the following day. Each monitoring session lasted 7–14 days (matching the sensor’s maximum wear duration). During monitoring, medical interventions such as dietary intake (carbohydrate consumption per meal) and insulin injection doses (rapid-acting/long-acting) were recorded, reflecting more complex blood glucose fluctuations in patients. A single fingerstick blood glucose measurement (gold standard) was collected before breakfast daily for CGM data calibration. Post-calibration data achieved MARD < 10%, meeting clinical requirements. Through variable screening, this study incorporated sensor_k, WE1, and Battery Voltage as input features. The sensor performance dimensions listed in Table 1—sensor_r (electrode linearity coefficient), sensor_k (electrode reaction rate), WE1 (working current), WE2 (contrast current), and Battery Voltage—are not present in the public dataset. Table 2 presents the statistical characteristics of the subjects.

### 3.2. Variable Selection

When training the blood glucose prediction model proposed in this paper, blood glucose levels are undoubtedly selected as the sole prediction target. However, we know that human blood glucose levels are influenced by multiple factors, and the interrelationships among these factors are highly complex. If only blood glucose values are used as input features for model training, the model will fail to learn the characteristics of other variables affecting blood glucose levels. Even if the predicted results appear satisfactory, the model will lack interpretability. Furthermore, selecting only one variable other than blood glucose as an input feature would prevent the model from learning the intricate relationships among all variables affecting blood glucose fluctuations. This would not only diminish the model’s predictive accuracy but also limit its applicability to predicting blood glucose levels for a specific subset of individuals. Therefore, to enhance both interpretability and generalization, it is necessary to screen all features listed in Table 1 and select multiple covariates as input features for the model.

**Figure 4:**
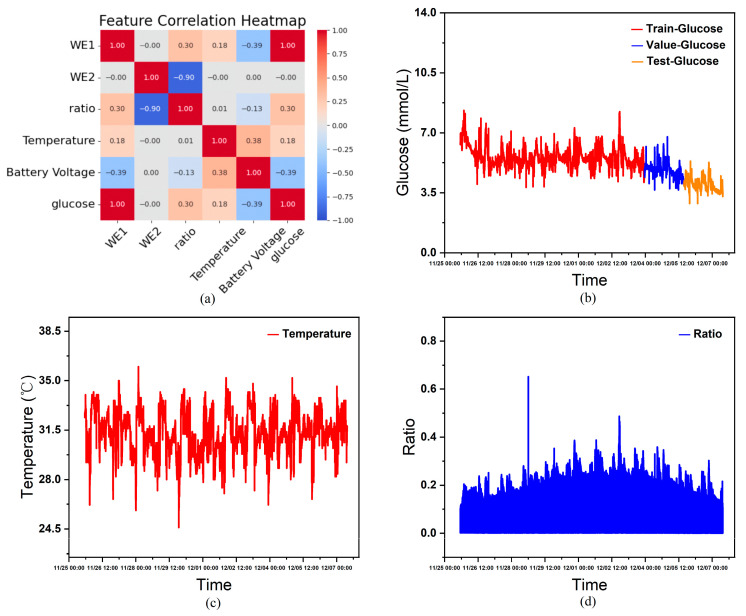
(**a**) Correlation heatmap between different features. (**b**) Visual display of divided dataset. (**c**) Temperature versus time curve. (**d**) Ratio of working current to blank current versus time curve.

Performing correlation heatmap analysis using the features in Table 1 as variables, this study excluded constant variables from the analysis since they exert minimal influence on other variables within the single-subject dataset. As shown in Figure 4a, the correlation heatmap derived from statistical analysis indicates that, except for WE2, all non-constant variables exhibit a certain degree of correlation with blood glucose levels. Furthermore, considering that features such as age, gender, and diabetes type significantly influence human blood glucose fluctuations, although these constant variables cannot aid model training in an individual’s continuous glucose data, when combining continuous glucose data from multiple individuals as the training set, the actual circumstances and CGM devices worn by each person vary. Inputting these characteristics helps the model learn the complex relationships between them and blood glucose fluctuations, thereby influencing the model’s prediction outcomes. Therefore, this paper ultimately selects age, sex (0 = male, 1 = female), type (0 = healthy, 1 = Type 1 diabetes, 2 = Type 2 diabetes), sensor_k, WE1 (nA), Temperature (°C), and Battery Voltage (V) as covariate input features, glucose (mmol/L) as the prediction target, and time as the temporal index. Figure 4c and Figure 4d show the curves of temperature and current ratio over time, respectively.

### 3.3. Data Preprocessing

The collected data, being real clinical data, are susceptible to various objective and subjective factors, such as issues in data recording and transmission, calibration algorithm errors, or human instability. Therefore, the presence of missing values and outliers in the data sample is unavoidable. Regarding missing values, since the model performs time-series predictions, omitting missing values also results in temporal gaps. This omission adversely affects model training. Considering that human blood glucose levels do not undergo drastic changes within short timeframes, this paper employs forward imputation to fill missing values. Considering that overly smoothed blood glucose time-series data may overlook hypoglycemic and hyperglycemic events, while directly learning from completely unfiltered data may lead to inaccurate model predictions, handling outliers requires a balance between these two extremes. Therefore, this paper adopts a filtering approach to process the raw data for outliers, employing a low-pass filter to eliminate outliers from the original data.

Specifically, among 25 patients, 3 cases (1 with type 1 diabetes and 2 with type 2 diabetes) had missing data, resulting in a missing rate of 5.2–7.8% (remaining patients had a missing rate < 2%); Missing values were imputed using the previous value method. After applying a 5 Hz low-pass filter, data completeness reached 98.3%. Outliers (exceeding the 2.8–22.2 mmol/L range) constituted < 0.5% and were excluded. Both processing methods preserved the characteristics of the original blood glucose data while enhancing its smoothness and accuracy to some extent. Inputting this processed data into the model improved the performance of the blood glucose prediction model.

The raw data for each sample is collected with millisecond precision. However, since hospitals primarily receive data via Bluetooth and local area networks, transmission errors inevitably occur during this process, resulting in variations in the time intervals between samples. Given the stringent requirement for temporal continuity in time-series analysis, coupled with significant differences in the data provided by different users, and the fact that each sensor is worn for no longer than 14 days, this study adopts a sampling frequency of one minute. This decision is grounded in practical considerations: human blood glucose levels do not undergo substantial fluctuations within second-long intervals.

Due to variations in patient age, diabetes type, and differences in CGM device sensitivity (sensor_k) and working current (WE1), this study integrates multi-patient data to train a global model. This approach enables the model to learn associations between individual differences and glucose fluctuations, thereby enhancing its generalization capability. For the preprocessed data, the first 70% is used as the training set for the model. Half of the remaining 30% is allocated as the validation set for model training, while the other half serves as the test set after training completion. Figure 4b illustrates the partitioned continuous glucose data.

### 3.4. Model Training

Reference [31] only performs supervised training on the final output layer without constraining intermediate layer features, which may cause the temporal characteristics learned in intermediate layers to deviate from physiological glucose patterns (e.g., predicting blood glucose values beyond clinically reasonable ranges). Therefore, an “intermediate supervision loss” (MAE loss, weighted at 0.3) is added after the first layer output in the dual-layer SCINet, with the final output layer loss weighted at 0.7. This ensures that the short-term features extracted by the first layer align with physiological blood glucose fluctuations (e.g., postprandial blood glucose increase within 15 min not exceeding 5 mmol/L). The total loss function is as follows:

$$TotalLoss=0.3\;\times {MAE}_{Layer1}+0.7\;\times {MAE}_{Final}$$

During training, MAE demonstrates greater robustness to outliers in blood glucose data (such as sudden hyperglycemia), preventing extreme values from unduly disrupting model training. It optimizes model parameters by minimizing the loss function. Concurrently, MARD—a commonly used clinical metric mentioned in the introduction—serves as one of the evaluation indicators. Together with MAE, R^2^, and accuracy, it is employed in the results analysis:


MAE =1n∑i=1  n|yi−y^i| (Measure absolute error);

$$MARD=\frac{1}{n}\sum_{i=1}^{n}\;\frac{\left|y_{i}-{\hat{y}}_{i}\right|}{y_{i}}\times 100\%\;(\mathrm{Measuring}\;\mathrm{relative}\;\mathrm{error});$$

$$R^{2}=1-\frac{\sum_{i=1}^{n}\;(y_{i}-{\hat{y}}_{i}{)}^{2}}{\sum_{i=1}^{n}\;(y_{i}-\bar{y}{)}^{2}}\;(\mathrm{Measuring}\;\mathrm{Model}\;\mathrm{Fit});$$

Accuracy rate: Percentage of samples with a difference between predicted and actual blood glucose values < 0.5 mmol/L.

In this case, n is the number of samples, $y_{i}$ is the actual value, and y^i is the predicted value.

The model makes predictions on the validation set and, through multiple iterations, progressively reduces the error between predicted and actual values to bring the predictions closer to the true blood glucose concentrations. The Adam optimizer is employed to update model parameters, enhancing training efficiency and model performance. An early stopping strategy is implemented during training: training ceases when the loss function fails to decrease for 100 consecutive epochs on the validation set, thereby preventing model overfitting. Finally, MARD, R^2^, accuracy, and MAE are collectively employed for result analysis.

## 4. Results and Discussion

**Figure 5:**
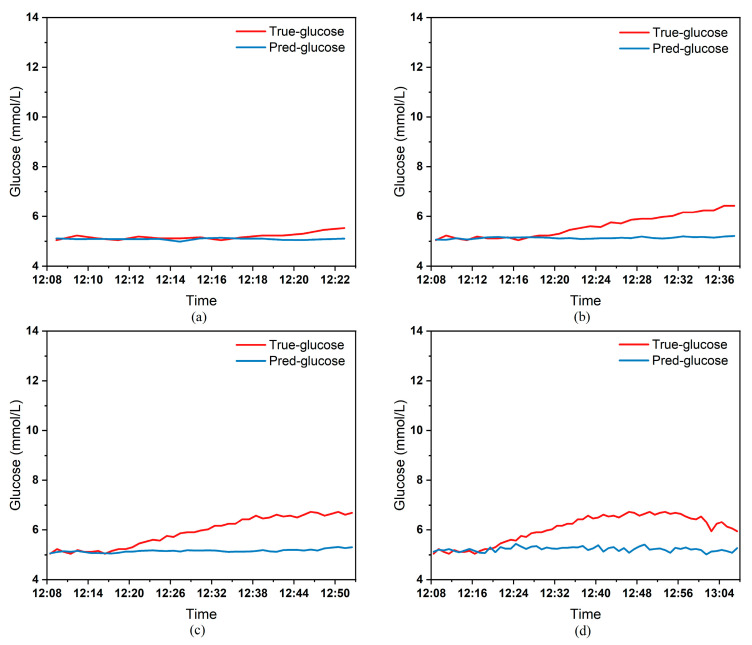
Plot of predicted versus true blood glucose curves for patient 16 after different prediction durations: (**a**) 15 min. (**b**) 30 min. (**c**) 45 min. (**d**) 60 min.

**Figure 6:**
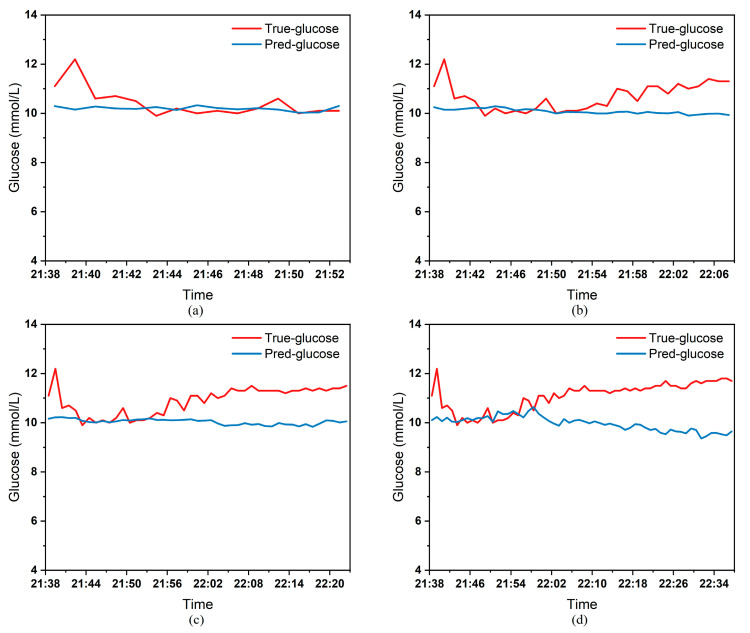
Plot of predicted versus true blood glucose curves for patient 18 after different prediction durations: (**a**) 15 min. (**b**) 30 min. (**c**) 45 min. (**d**) 60 min.

**Figure 7:**
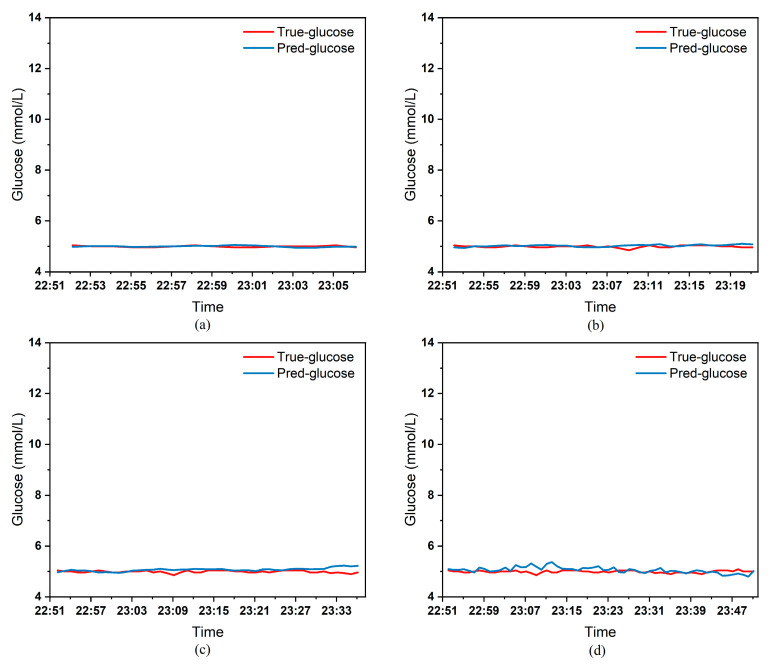
Plot of predicted versus true blood glucose curves for patient 20 after different prediction durations: (**a**) 15 min. (**b**) 30 min. (**c**) 45 min. (**d**) 60 min.

**Figure 8:**
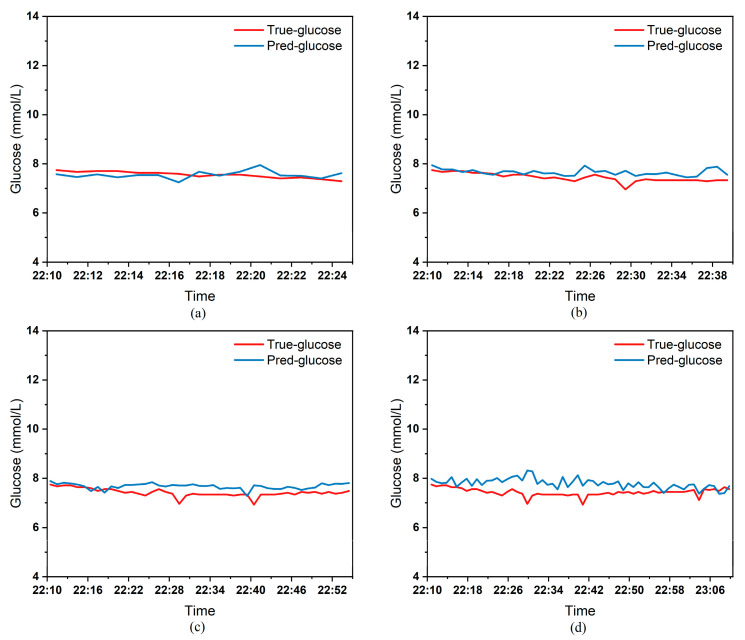
Plot of predicted versus true blood glucose curves for patient 22 after different prediction durations: (**a**) 15 min. (**b**) 30 min. (**c**) 45 min. (**d**) 60 min.

**Figure 9:**
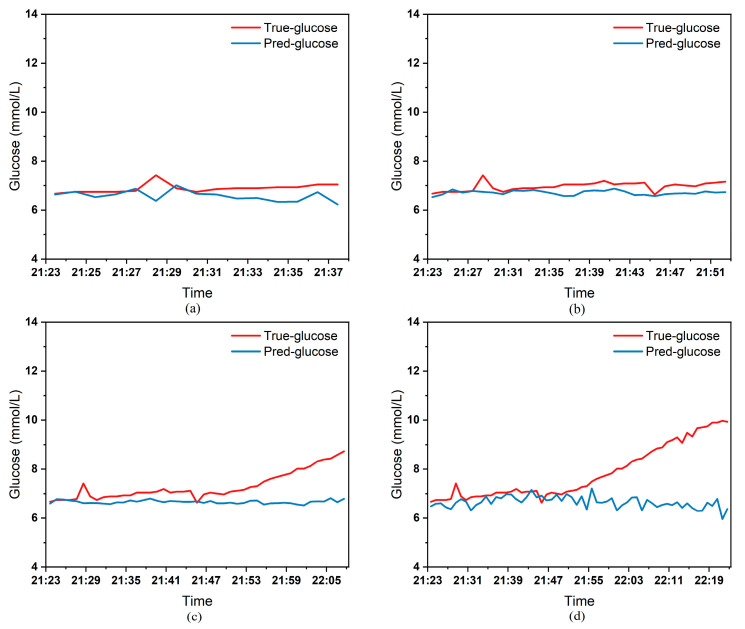
Plot of predicted versus true blood glucose curves for patient 24 after different prediction durations: (**a**) 15 min. (**b**) 30 min. (**c**) 45 min. (**d**) 60 min.

To clearly demonstrate the performance of the proposed model in blood glucose prediction tasks, this paper selected five diabetic patients and plotted comparison graphs of predicted versus actual blood glucose values across different prediction time horizons. Figure 5, Figure 6, Figure 7, Figure 8 and Figure 9, respectively, show the comparison between the predicted glucose curves and the actual glucose curves for patients 16, 18, 20, 22, and 24 over the next 15 min, 30 min, 45 min, and 60 min. The horizontal axis represents time, and the vertical axis represents glucose levels (mmol/L). The figures reveal that within the 15 min prediction window, the model’s predicted curve most closely aligns with the patient’s actual glucose curve. As the prediction time extends, a time-lag error begins to emerge between predicted and actual values, with prediction accuracy gradually declining. By the 60 min prediction window, the model’s predicted curve exhibits the poorest fit with the patient’s actual glucose curve.

The model also exhibits varying performance across different patient datasets. As shown in Figure 5, Figure 6, Figure 7, Figure 8 and Figure 9, the fitting accuracy between the predicted glucose curves and actual glucose curves differs significantly for these five patients under identical prediction durations. For instance, under the same prediction period, patients 18 (Figure 6) and 24 (Figure 9) demonstrate substantial lag errors between predicted and actual values, indicating poor alignment between the predicted and actual glucose curves. In contrast, Patient 20 (Figure 7) exhibits consistently good alignment between the predicted and actual glucose curves across all prediction durations. Macro-level analysis reveals that the overall trends of glucose prediction curves and actual glucose curves are largely consistent across patients. Although micro-level deviations exist, the absolute error between the model’s predicted glucose values and actual values at each time point is not substantial.

Table 3 presents the Mean Absolute Error (MAE) between the model’s predicted blood glucose values and actual values for multiple patients across different prediction durations. In fact, as the prediction duration increases, factors influencing blood glucose fluctuations become more complex and difficult to predict. The model struggles to accurately capture long-term fluctuation trends, leading to a gradual increase in the error between predicted and actual values. Table 3 clearly demonstrates that within a 15 min prediction horizon, the mean MAE between predicted and actual blood glucose values across multiple patients was only 0.2365 mmol/L—the smallest error among the four prediction horizons. In contrast, at the 60 min prediction horizon, the mean MAE reached 0.9376 mmol/L. Compared to the 15 min prediction results, this indicates that the model achieves superior prediction accuracy within shorter forecasting periods.

Additionally, variations in prediction accuracy across different patients may stem from multiple factors. On one hand, each patient exhibits distinct patterns of blood glucose fluctuations, with some experiencing significantly greater amplitude and frequency of fluctuations than others, which undoubtedly increases predictive complexity. On the other hand, constrained by device limitations and human operational factors, the quality of raw data collected from different patients in this study also varies considerably. A small number of patients exhibited relatively high rates of missing glucose data, which adversely affected the time-series prediction task. These variations in data quality caused the same model to demonstrate differing performance across different patients’ datasets. Table 3 reveals that across the four prediction time horizons, Patient 19 exhibited the worst model performance among all patients, with a mean MAE of 2.1826 mmol/L between predicted and actual blood glucose values. Conversely, Patient 20 demonstrated excellent model performance, achieving a mean MAE of only 0.0729 mmol/L. Overall, however, the MAE between predicted and actual blood glucose values for each patient across different prediction time horizons was generally maintained below 1.0 mmol/L, sufficiently demonstrating the model’s reliability for blood glucose prediction tasks. (Annotation: True value refers to CGM-calibrated blood glucose, validated by daily fingertip blood tests MARD < 10%.)

Table 4 presents the mean values of the model evaluation metrics mentioned in Section 3.4, further demonstrating the superiority of the model designed in this paper. To avoid patient identity disclosure, the personalized model was tested on 5 anonymized patients (1 with Type 1 diabetes, 4 with Type 2 diabetes) whose demographic characteristics are consistent with the overall cohort (Table 2). Their age, BMI, and monitoring duration fall within the 25th–75th percentiles of the total subjects, ensuring representativeness (Their anonymized demographic data are as follows: age range 42–68 years (mean 55.2 ± 9.8), BMI range 23.1–27.8 kg/m^2^ (mean 25.3 ± 1.6), average monitoring duration 9.5 ± 1.8 days. These patients were chosen to cover diverse glucose fluctuation patterns (mild/moderate/severe) to verify generalizability. Table 5 shows the results of the personalized model comparison experiments. The personalized model exhibits superior performance across all prediction durations but requires separate training for each patient. In clinical applications, a trade-off must be made between “prediction accuracy” and “training efficiency.”

The performance improvement of the personalized model is not an isolated case. For the remaining 5 diabetic patients not explicitly shown in Table 5, the personalized model also reduced the MAE by 15.2–17.5% (15 min: 0.201–0.228 mmol/L → 0.168–0.191 mmol/L; 60 min: 0.892–1.056 mmol/L → 0.735–0.884 mmol/L) and MARD by 0.8–1.6% compared to the global model. This consistency confirms that the personalized model’s advantage in capturing patient-specific glucose patterns is generalizable across diverse patients, rather than limited to individual cases.

This paper also conducted comparative experiments on multiple time-series prediction models, including both AI and non-AI models. The experiments compared the blood glucose prediction results of these models across different time horizons. Table 6 presents the mean absolute error (MAE) between the predicted and actual blood glucose levels for multiple patients over the next 15, 30, 45, and 60 min. Based on the experimental results presented in Table 6, the MAE of the model designed in this paper (DS-SCINet) is significantly lower than that of non-AI models, validating the advantages of AI models in blood glucose prediction. Additionally, it can be observed that within the 15 min prediction window, the performance of the blood glucose prediction models based on CNNLSTM and TCN is comparable to that of the model designed in this paper. However, as the prediction time increases, the model designed in this paper continues to maintain a smaller prediction error. This indicates that the model can still fully capture the complex relationship between blood glucose fluctuations and various factors affecting blood glucose changes over longer prediction periods, demonstrating the superiority of the proposed model in terms of prediction accuracy.

The 0.4 mmol/L reduction in MAE (60 min prediction: Kalman filter 1.3254 mmol/L vs. DS-SCINet 0.9376 mmol/L, Table 6) has tangible clinical significance, particularly in scenarios where precise glucose prediction guides intervention decisions.

First, in hypoglycemia warning (a critical clinical priority), suppose the hospital starts intervention when the patient’s blood glucose is < 3.9 mmol/L. If the actual glucose is 3.5 mmol/L (hypoglycemic), the Kalman filter may predict 3.9 mmol/L (error + 0.4 mmol/L)—failing to trigger timely intervention—while DS-SCINet predicts 3.5 mmol/L (accurate), enabling early action (e.g., consuming 15 g carbohydrates) to prevent severe hypoglycemia (≤3.0 mmol/L), which is associated with seizures or cognitive impairment.

Second, in target glucose control for vulnerable populations (e.g., critically ill patients, pregnant women with gestational diabetes). A 0.4 mmol/L error could misclassify glucose status: if actual glucose is 7.4 mmol/L (mild hyperglycemia), the Kalman filter may predict 7.0 mmol/L (within target, no intervention), while DS-SCINet predicts 7.4 mmol/L (triggering insulin dose adjustment). This avoids delayed correction of hyperglycemia, which is linked to increased infection risk and longer hospital stays. For patients with stable glucose (e.g., 5.0 mmol/L), the 0.4 mmol/L error (5.0 vs. 5.4 mmol/L) may not require immediate intervention, but consistent small errors can accumulate over 6–12 h, leading to suboptimal long-term glycemic control (e.g., HbA1c elevation).

Limitations should be noted: this study’s clinical impact is inferred from error reduction and existing guidelines, not prospective clinical trials. Future work will validate DS-SCINet in a randomized controlled trial (RCT) to measure changes in intervention frequency, hypoglycemia/hyperglycemia incidence, and patient-reported outcomes (e.g., quality of life).

## 5. Conclusions

This paper introduces a blood glucose prediction model constructed using SCINet. While detailing the model design process, theoretical analysis clarifies SCINet’s significant advantages in handling time-series data. Subsequently, the SCINet architecture undergoes further refinement, leading to the design of a blood glucose prediction model based on a stacked dual-layer SCINet. This enhancement significantly boosts the model’s capability to learn complex nonlinear features in blood glucose data. To validate the proposed model’s performance, continuous glucose monitoring data from real patients at Yixing People’s Hospital were used as the experimental dataset. The dataset was divided into training, validation, and test sets. The model was trained using the training set, with optimal hyperparameters selected through iterative experiments on the validation set. Model evaluation was conducted on the test set. Specifically, correlation experiments were conducted to screen human characteristics captured by CGM sensors, identifying key features influencing blood glucose fluctuations. The selected input features for the model include age, gender, diabetes type, sensor sensitivity, sensor operating current, temperature, battery voltage, and calibrated blood glucose value. Subsequently, various anomalies present in the raw data were appropriately addressed. Next, the model training strategy was briefly outlined. Finally, model performance was evaluated on the test dataset. The experimental results demonstrate that the proposed blood glucose prediction model maintains low prediction errors when forecasting both time-varying glucose fluctuations and glucose changes across different patients. Furthermore, comparative experiments with existing time-series prediction models on the same dataset conclusively show that the proposed model outperforms existing models in both prediction accuracy and stability.

## Figures and Tables

**Table 1:** Data features that sensors can collect.

Variable	Effect
time	Indicates the exact time when these data were collected
patient_id	Indicates the patient’s number
age	Indicates the patient’s age
sex	Indicates the patient’s sex
type	Indicates the type of diabetes the patient has (Type I, Type II, or Healthy)
sensor_r	Indicates the linear correlation coefficient of the electrode response in a gradient glucose solution
sensor_k	Indicates the rate at which the electrode reacts in a glucose gradient solution
needle_id	Indicates the number of the needle inside the sensor
sensor_id	Indicates the sensor number
WE1	Indicates the working current generated by the reaction between tissue fluid and the enzyme
WE2	Indicates the contrast current generated on the electrode without the enzyme
ratio	Indicates the ratio of working current to blank current
Temperature	Indicates the temperature when these data were collected
Battery Voltage	Indicates the battery voltage when these data were collected
glucose	Indicates the calibrated blood sugar value

**Table 2:** Subject demographic characteristics table.

Feature	Value (n = 25)
Total number of subjects	25 (id: 0–24)
Type of diabetes (n, %)	Healthy population: 15 (id: 0–14) cases (60%)
	Type 1 diabetes: 1 (id: 15) cases (4%)
	Type 2 diabetes: 9 (id: 16–24) cases (36%)
Age (years, x ± s)	56.3 ± 12.7 (range: 28-78)
Sex (n, %)	Male: 15 cases (60%)
	Female: 10 cases (40%)
BMI (kg/m^2^, x ± s)	25.1 ± 3.2 (range: 20.3–31.5)
Average monitoring days (days)	9.2 ± 2.1
Average daily sampling points	225 ± 25

**Table 3:** MAE of model across different prediction horizons.

Patient	15 min	30 min	45 min	60 min	Average Value
15	0.1092	0.1094	0.2026	0.1992	0.1551
16	0.1384	0.4789	0.7822	0.8398	0.5598
17	0.1905	0.2456	0.2566	0.2597	0.2381
18	0.3837	0.6406	0.8680	1.1578	0.7625
19	0.7102	1.3845	2.3070	4.3287	2.1826
20	0.0357	0.0573	0.0883	0.1102	0.0729
21	0.2243	0.2425	0.4064	0.6819	0.3888
22	0.1778	0.2158	0.2687	0.3691	0.2579
23	0.0601	0.1091	0.1985	0.2268	0.1486
24	0.3355	0.2626	0.6419	1.2029	0.6107
Average value	0.2365	0.3746	0.6020	0.9376	0.5377

**Table 4:** Mean value of model evaluation indicators.

Patient	MAE (mmol/L)	MARD (%)	R^2^	Accuracy (%)
15	0.1551	8.2	0.96	94.3
16	0.5598	10.5	0.87	82.6
17	0.2381	8.7	0.95	92.1
18	0.7625	12.3	0.81	76.8
19	2.1826	14.8	0.72	68.5
20	0.0729	7.9	0.98	96.7
21	0.3888	9.6	0.91	88.4
22	0.2579	8.9	0.94	91.5
23	0.1486	8.1	0.97	95.2
24	0.6107	11.2	0.85	80.3
Average value	0.5377	10.0	0.90	86.6

**Table 5:** Comparison experiment results of personalized models.

Model Type	Prediction Duration	MAE (mmol/L)	MARD (%)	R^2^
Global Model	15 min	0.2365	8.2	0.92
Personalized model	15 min	0.1987	7.5	0.95
Global Model	30 min	0.3746	9.5	0.88
Personalized model	30 min	0.3142	8.7	0.92
Global Model	45 min	0.6020	11.0	0.83
Personalized model	45 min	0.4935	9.2	0.89
Global Model	60 min	0.9376	12.1	0.81
Personalized model	60 min	0.7852	10.3	0.86

**Table 6:** Model Performance Comparison of Multiple Timing Prediction Algorithms.

Model	The Mean Value of MAE			
	15 min	30 min	45 min	60 min
Kalman filter	0.3124	0.5218	0.9876	1.3254
IIR filter	0.3567	0.5892	1.0543	1.4127
CNNLSTM	0.2481	0.4837	0.8974	1.1835
TCN	0.2573	0.5351	1.1020	1.7725
Transformer	2.7262	2.7338	2.7859	2.8223
Informer	6.1508	6.2682	6.2556	6.4504
SCINet	0.2577	0.4019	0.9132	0.9956
DS-SCINet	0.2365	0.3746	0.6020	0.9376

## Data Availability

The data presented in this study are available on request from the corresponding author due to (involving privacy).
